# Dual Stream Transmission and Downlink Power Control for Multiple LEO Satellites-Assisted IoT Networks

**DOI:** 10.3390/s22166050

**Published:** 2022-08-12

**Authors:** Bingyu Xu, Xiayu Li, Yujuan Ma, Xing Xin, Michel Kadoch

**Affiliations:** 1 China Academy of Information and Communications Technology, Beijing 100191, China; 2 Innovation Center for Satellite Communication System, China National Space Administration, Beijing 100191, China; 3 School of Electronic Engineering, Beijing University of Posts and Telecommunications, Beijing 100876, China; 4 Ecole de Technologie Supérieure, Montreal, QC H3C 1K3, Canada

**Keywords:** dual-satellite system, power control, synchronization, LEO satellite

## Abstract

The multi-satellites cooperative transmission can effectively increase the data rate that can be achieved by internet of things (IoT) terminals. However, the dynamic characteristics brought by low Earth orbit (LEO) satellites will seriously decrease the data rate and make the data rate fluctuate. In this paper, dual-stream transmission and downlink power control for multiple LEO satellites-assisted IoT networks are investigated. To mitigate the effects of the frequency offset caused by different LEO satellites, a multi-satellites synchronization scheme is proposed. Then, different power control schemes are given to resist the data rate fluctuation during the transmission. The simulation results show that the proposed schemes can effectively compensate for the varied frequency offset and keep the data rate stable.

## 1. Introduction

The sixth-generation (6G) wireless networks will be a space–air–ground–sea integrated network [1,2,3], where satellite communication plays an important role in providing seamless coverage globally. Especially for internet of things (IoT) networks, which are usually deployed in remote areas or at sea, satellite communication is an indispensable component that can provide service at any time. For example, in the industrial internet of things (IIoT) scenario, IoT terminals usually support a large number of geographically dispersed nodes and are located in harsh environments, such as open seas, dangerous areas, mountains, etc., so it is difficult for the ground network to cover [4,5]. Because the satellite communication system has the characteristics of wide coverage and strong survivability, the satellite-assisted IoT (S-IoT) can realize wide area coverage, remote information transmission and acquisition. Therefore, S-IoT networks have received lots of attention recently. Traditional S-IoT focuses on the narrow-band IoT services, but it cannot meet the requirement of the emerging requirements for IoT services, such as high-precision object recognition. Moreover, more and more of the deployed IoT networks will also need to feed the aggregated data back to the control center, and higher system throughput is needed to bear a large amount of IoT data. Unfortunately, the capacity or data transmit rate achieved by a single satellite is usually limited by the high path loss and the scarce resources available on the satellites. In order to achieve a higher data rate, cooperative transmission with multiple satellites has been seen as a feasible method to effectively improve the system throughput.

The multi-satellite cooperative transmission can effectively improve the spectral efficiency and communication rate. A multi-satellites cooperation system composed of two geostationary Earth orbit (GEO) satellites was proposed in [6,7]. The satellites adopted zero-forcing (ZF) coding and carried out the power allocation according to the maximization of the data rate under per-antenna power (PAC) constraint, and the cooperative scheme increased the spectral efficiency compared to the conventional techniques. In [8], the cooperative transmission strategy of the GEO satellite colocation (GEOSC) system was studied. The multipath component and the line-of-sight (LOS) component in the Rician channel were comprehensively considered to improve the transmission rate of the system through the user selection strategy and opportunistic beamforming. In [9], the cooperative transmission strategy for the multi-satellite colocation (MSC) system was proposed, which adopted the cooperative beamforming and constructed an orthogonal channel by geometric criterion. The communication rate was better than the two-satellite GEOSC system. A full frequency reuse-based dual-satellite system was studied in [10] to achieve a high data transmission rate by designing a relay-aided decision support system. In recent years, with the development of LEO satellite techniques, the cooperative techniques between GEO and LEO satellites have attracted attention. The multi-satellites relay systems based on time division multiple access (TDMA) and non-orthogonal multiple access (NOMA) were introduced in [11]. In [12,13], spectrum sharing and cooperative technology were considered in the LEO-GEO co-existing systems, which can solve the interference issue between different satellites. In addition, a cooperative LEO satellites system was studied in [14,15]. In [14], the effect of different antenna distributions with multiple antenna terminals in a dual-LEO-satellite system was analyzed. In [15], the ultra-dense LEO satellite constellation networks were considered, and the algorithm based on the max–min fairness criterion and analog beamforming was proposed to improve the system capacity.

Due to large path loss, power control is especially important in satellite systems. In [16], the application of Q-learning for power control was investigated in the satellite communication system, and the proposed method effectively extended the service life of the satellite battery. Considering the distributed power control problem in downlink cognitive satellite–terrestrial networks, a static non-cooperative game model was proposed in [17] to improve the resource utilization efficiency and reduce interference. The authors in [18] studied the LEO satellite constellation model under the dynamic characteristics of an LEO satellite. Aiming to limit the maximum delay and minimize the outage probability, two optimal power control schemes were proposed. In order to improve the performance of the uplink random access, a power control scheme based on spatial grouping was proposed in [19], which effectively reduced the energy consumption of satellite IoT terminals. In [20], the pilot-based channel estimation and back-off interference power constraint were employed for the satellite link and the terrestrial interference link, respectively, and the proposed method can alleviate the impact of imperfect channel station information.

However, the above research works related to cooperative satellite systems did not consider the large frequency offset caused by LEO satellites. In the LEO satellite system, the synchronization performance is seriously affected by the large frequency shift caused by the high-speed movement of the LEO satellites. How to carry out accurate synchronization under a large frequency shift is an important problem. There have been plenty of research works on the subject of synchronization for the terrestrial communications system. Low-complexity synchronization schemes were proposed in [21,22] by using the autocorrelation of primary synchronization signals (PSS) for a 5G system. These works reduced the complexity by sacrificing the SNR performance or processing delay. The work in [23] exploited the periodicity and the sparsity of the channel to improve the estimated performance of the channel impulse response (CIR) and of the carrier frequency offset (CFO). Most of the above research works are aimed at reducing the complexity, and they are not applicable to the high mobility of LEO satellites. In an LEO satellite system, the large frequency offset seriously affects the system performance. To achieve fine timing synchronization, a low-complexity algorithm using the Zadoff–Chu (ZC) sequence is proposed in [24]. The work in [25] proposed a robustness timing advance estimation method by a novel random-access preamble sequence, which consists of the real and imaginary part of a ZC sequence. The time and frequency synchronization methods in the downlink LEO satellite system were investigated in [26]. The PSS and cyclic prefix (CP) were used to improve the estimation performance. In order to adapt to 5G, the synchronization methods suitable for LEO satellite single-path and multipath fading channels were proposed in [27] for an OFDM-based satellite system, which utilizes the ZC sequence and the feature of the satellite channel. In addition, several methods estimated the frequency offset without training sequences. In [28], the author presented a turbo-code-aided Doppler frequency shift correction algorithm by means of the Gaussian process model for high-mobility satellite communication without a training sequence.

**Figure 1:**
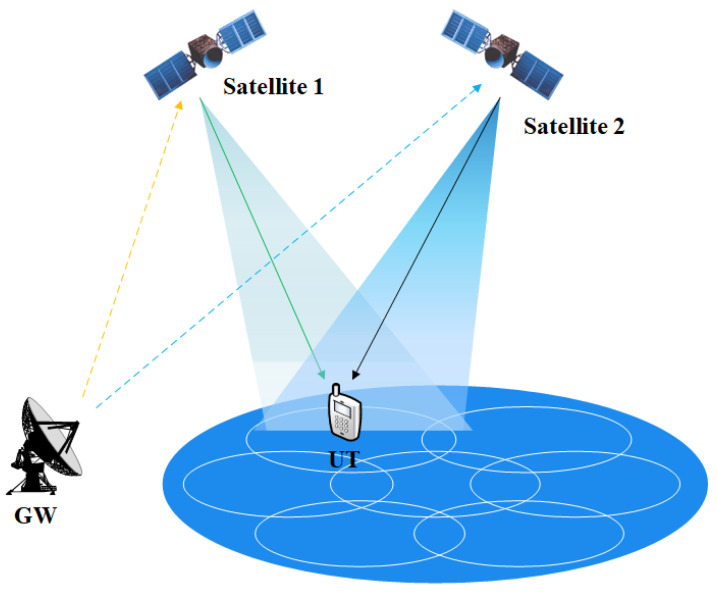
The dual-stream transmission for LEO satellites-assisted IoT network.

In this paper, we model the downlink transmission of a dual-LEO-satellite communication system and analyze the impact of the dynamic characteristics of an LEO satellite. In addition, we propose a power control algorithm and a synchronization algorithm under this system to combat the influence of the path loss and frequency offset of the LEO satellite system. It is assumed that the data transmitted by the two satellites are different, and the terminal has the function of joint receiving and processing of the dual-stream data, as shown in Figure 1. A power control scheme is proposed to solve the path loss effect. In order to resist the large frequency shift of the LEO satellites, the two-dimensional search algorithm is adopted. The PSS sequence is used for the time synchronization and coarse frequency offset estimation, and the PSS and secondary synchronization signal (SSS) sequences are combined for the fine frequency offset estimation to improve the synchronization performance. The simulation results show that the proposed schemes can not only ensure a high communication rate but also reduce the power loss. The proposed scheme can achieve more than a 1 Gbps data rate for a single user.

## 2. System Model

### 2.1. Dual-LEO-Satellite System Model

Considering the cooperative transmission of dual-satellite communication system, there are two satellites that transmit data to the single IoT user terminal (UT). Each satellite is equipped with a planer antenna array containing antenna elements. The user terminal is equipped with two reflector antennas. The signals received by terminal’s antenna 1 and antenna 2 at a certain time can be expressed as

(1) $$\begin{array}{c} y_{1}\left(n\right)=G_{R1}G_{T1}h_{1,1}w_{1}x_{1}\left(n\right)+G_{R2}G_{T1}h_{1,2}w_{2}x_{2}\left(n\right)+z_{1}\left(n\right) \end{array}$$

(2) $$\begin{array}{c} y_{2}\left(n\right)=G_{R1}G_{T2}h_{2,1}w_{2}x_{2}\left(n\right)+G_{R2}G_{T2}h_{2,2}w_{1}x_{1}\left(n\right)+z_{2}\left(n\right) \end{array}$$

where $G_{T1}$ and $G_{T2}$ are the antenna gain of satellite 1 and satellite 2. $G_{R1}$ and $G_{R2}$ are the antenna gain of the terminal’s antenna 1 and terminal’s antenna 2, respectively. $h_{1,1}$, $h_{1,2}$, $h_{2,1}$, $h_{2,2}\in C^{1\times N_{t}}$ represent the channel response. $w_{1}$, $w_{2}\in C^{N_{t}\times 1}$ denote analog beamforming weighting vectors of satellite 1 and satellite 2. $x_{1}\left(n\right)$, $x_{2}\left(n\right)$ denote the transmit signal of satellite 1 and satellite 2. $z_{1}\left(n\right)$, $z_{2}\left(n\right)$ represent the additive noise of the terminal’s antenna 1 and antenna 2. $y_{1}\left(n\right)$, $y_{2}\left(n\right)$ denote the receive signal of antenna 1 and antenna 2, respectively. Note that $G_{R1}G_{T1}h_{1,1}w_{1}x_{1}\left(n\right)$ in (1) denotes signal from satellite 1 received by antenna 1, and $G_{R2}G_{T1}h_{1,2}w_{2}x_{2}\left(n\right)$ represents the signal from satellite 2 received by antenna 1. $G_{R1}G_{T2}h_{2,1}w_{2}x_{2}\left(n\right)$ denotes signal from satellite 2 received by antenna 2, and $G_{R2}G_{T2}h_{2,2}w_{1}x_{1}\left(n\right)$ represents the signal from satellite 1 received by antenna 2.

**Figure 2:**
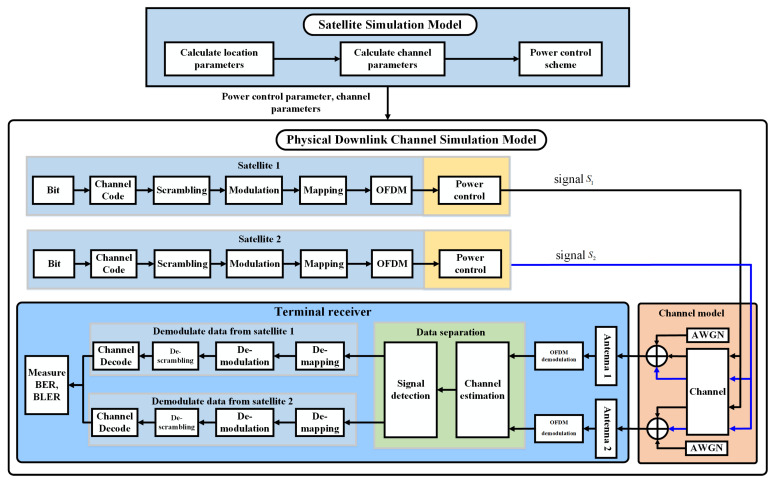
System simulation model.

To evaluate the performance of the dual-stream transmission with two LEO satellites, we set up a simulation platform. As shown in Figure 2, the system simulation model mainly includes two parts: the satellite simulation model and the downlink physical channel simulation model. The satellite simulation model includes satellite orbit model and power control module. The satellite orbit model mainly models the satellite orbit according to the satellite simulation parameters and obtains the position parameters of two satellites at any time. Then, calculate the relative position of ground users and satellites. The power control module mainly realizes power optimization by controlling power parameters. Downlink physical channel simulation includes signal transmitters, channel, signal receiver, bit error rate(BER) statistics and other modules to verify the system performance.

Satellite model simulation can be divided into three parts. First, according to the six orbital elements of satellite, simulate the satellite orbit, which can calculate the location parameters, such as the distance between the satellite and the user, the angle of arrival and angle of elevation. Second, calculate the parameters required by the channel model through the position parameters. Third, the power optimization scheme is adopted to adjust the power control parameters. Finally, the channel model parameters and power control parameters are output to the physical downlink channel simulation model.

The physical downlink channel simulation model process is shown in Figure 2, which consists of transmitters, channel model and receiver. At the transmitter, two satellites send two different data streams. The receiver demodulates the data separately by two receiving antennas. Set the user’s two receiving antennas as antenna 1 and antenna 2. Assuming at the receiver, antenna 1 expects to receive the transmitted data from satellite 1, antenna 2 expects to receive the transmitted data from satellite 2. Satellite 1 and satellite 2 generate different data followed by encoding, scrambling, modulation and power control at the transmitter. At the receiver, the received signals from antenna 1 and antenna 2 are forwarded to the OFDM demodulation module. The data separation scheme is realized by channel estimation and signal detection. The linear-minimum-mean-square-error (LMMSE) estimation algorithm is used for channel estimation [29]. The signal detection scheme in this paper adopts the maximum likelihood (ML) method, and the ML detector can be expressed as [30]

(3) $$\begin{array}{c} \left({\hat{x}}_{1}^{g},{\hat{x}}_{2}^{g}\right)=\arg\min_{\left(x_{1}^{g},x_{2}^{g}\right)}\sum_{r=1}^{2}{∥y_{r}^{g}-\sum_{t=1}^{2}diag\left(x_{t}^{g}\right){\hat{h}}_{r,t}^{g}∥}^{2} \end{array}$$

Splitting the received OFDM block into *G* sub-blocks, where g∈{1,…,G} denotes the *g*-th sub-block of the received OFDM signal. $x_{t}^{g},t\in \left\{1,2\right\}$ represents the modulation symbol of the *g*-th sub block transmitted by the *t*-th satellite. $y_{r}^{g},r\in \left\{1,2\right\}$ denotes the received signal of the *g*-th sub-block received by the *r*-th receiving antenna. ${\hat{h}}_{r,t}^{g},r\in \left\{1,2\right\},t\in \left\{1,2\right\}$ represents the estimated channel response vector. ${\hat{x}}_{1}^{g},{\hat{x}}_{2}^{g}$ represent the estimated modulation symbols transmitted by satellite 1 and satellite 2, respectively. After that, the transmission symbols of satellite 1 and satellite 2 are demodulated and decoded, respectively. Finally, the communication rate of the system can be calculated by counting BER and bit block error rate (BLER).

### 2.2. Frame Structure

The frame structure adopts the 5G frame structure based on the 3GPP specifications [31]. The frame length is 10 ms, which consists of ten subframes of 1 ms duration. Each subframe contains $N_{slot}^{subframe,\mu }N_{symb}^{slot}$ OFDM symbols, where $N_{symb}^{slot}$ denotes number of OFDM symbols per slot. $N_{slot}^{subframe,\mu }=2^{\mu }$ represents the number of slots contained in each subframe. μ represents the subcarrier spacing configuration. In the frequency domain, a resource block contains 12 consecutive subcarriers. The subcarrier spacing can be represented as $\Delta f=2^{\mu }15$ [kHz].

### 2.3. Synchronization Sequence

**Figure 3:**
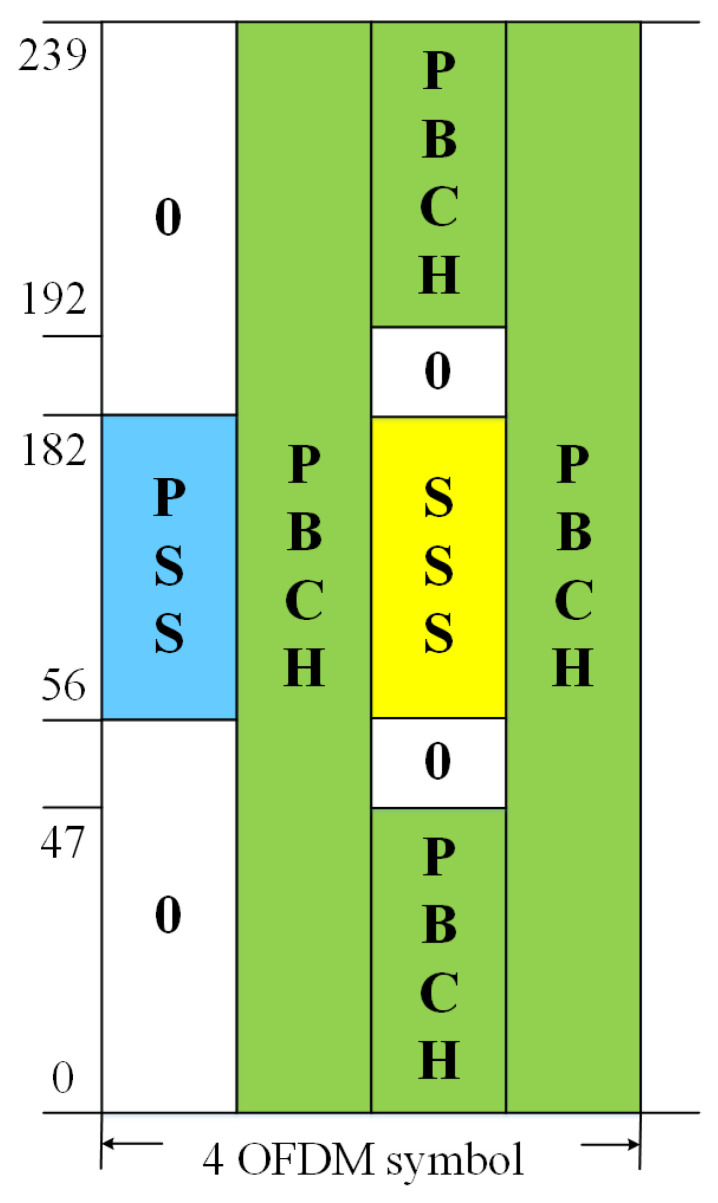
SS/PBCH resource mapping.

The synchronization scheme adopts the PSS and SSS. The PSS and SSS are located on SS/PBCH Block (SSB). The structure of SSB is shown in Figure 3. PSS and SSS occupy 127 subcarriers in frequency domain subcarriers in the frequency domain. The PSS sequence is defined by

(4) $$\begin{array}{c} d_{\mathrm{PSS}}\left(n\right)=1-2x\left(\left(n+43N_{\mathrm{ID}}^{\left(2\right)}\right)\;\mathrm{mod}\;127\right),\\ 0\leq n<127 \end{array}$$

(5) $$\begin{array}{c} x\left(i+7\right)=\left(x\left(i+4\right)+x\left(i\right)\right)\;\mathrm{mod}\;2 \end{array}$$

(6) $$\begin{array}{c} \left[x(6),x(5),x(4),x(3),x(2),x(1),x(0)]=[1110110\right] \end{array}$$

The SSS sequence is defined by

(7) $$\begin{array}{cc} \; & d_{\mathrm{SSS}}\left(n\right)=\left[1-2x_{0}\left(\left(n+m_{0}\right)\;\mathrm{mod}\;127\right)\right]\cdot \\ \; & \left[1-2x_{1}\left(\left(n+m_{1}\right)\;\mathrm{mod}\;127\right)\right]\\ \; & m_{0}=15\left\lfloor \frac{N_{\mathrm{ID}}^{\left(1\right)}}{112}\right\rfloor +5N_{\mathrm{ID}}^{\left(2\right)}m_{1}=N_{\mathrm{ID}}^{\left(1\right)}\;\mathrm{mod}\;112\\ \; & 0\leq n<127 \end{array}$$

where $N_{\mathrm{ID}}^{\left(1\right)}\in \{0,1,\ldots 335\}$, $N_{\mathrm{ID}}^{\left(2\right)}\in \left\{0,1,2\right\}$ represents the physical-layer cell identities.

### 2.4. Channel Models

In this section, we model the LEO satellite downlink channel [32]. $h_{l,k}\left(n\right)$ denotes the channel of satellite *l* and antenna *k*, which can be represented as

(8) $$h_{l,k}\left(n\right)=h_{l,k}^{LOS}\left(n\right)+h_{l,k}^{NLOS}\left(n\right)$$

where $h_{l,k}^{LOS}\left(n\right)$ represents the LOS part and $h_{l,k}^{NLOS}\left(n\right)$ is the none-line-of-sight (NLOS) part of the channel. They can be expressed as

(9) $$\begin{array}{c} h_{l,k}^{LOS}\left(n\right)=\sqrt{\frac{K_{l,k}}{K_{l,k}+1}}\exp\left[j2\pi \left(\varepsilon_{l,k}^{LOS}\frac{n}{N}-j2\pi f_{c}\tau_{l,k}^{LOS}\right)\right]v_{l,k}^{LOS} \end{array}$$

(10) $$\begin{array}{c} h_{l,k}^{NLOS}\left(n\right)=\sqrt{\frac{1}{K_{l,k}+1}}\sum_{m=1}^{M_{l,k}}g_{l,k,m}\exp\left[j2\pi \left(\varepsilon_{l,k,m}^{NLOS}\frac{n}{N}-j2\pi f_{c}\tau_{l,k,m}^{NLOS}\right)\right]v_{l,k}^{NLOS} \end{array}$$

where $K_{l,k}$ denotes the Rician factor. $\varepsilon_{l,k}^{LOS}=f_{l,k}^{LOS}/\Delta f$, $\varepsilon_{l,k,m}^{NLOS}=f_{l,k,m}^{NLOS}/\Delta f$ denote the normalized Doppler shift of the LOS and NLOS, respectively. Δf represents the subcarrier spacing. *N* denotes the Fourier transform points. $f_{c}$ is the carrier frequency. $\tau_{l,k}^{LOS}$ and $\tau_{l,k,m}^{NLOS}$ represent the propagation delay of the LOS and NLOS. $M_{l,k}$ denotes the number of channel propagation paths of NLOS. $v_{x,y}\overset{\Delta }{\;=}$$\left\{v_{l,k}^{LOS},v_{l,k}^{NLOS}\right\}$ denote the array response array. As the satellite is very high relative to the terrestrial terminal, the array response $v_{l,k}^{LOS}=v_{l,k,m}^{NLOS}$, which can be expressed as

(11) $$\begin{array}{c} v_{x,y}=v^{x}⊗v^{y}\in C^{1\times M} \end{array}$$

(12) $$\begin{array}{cc} v^{x}= & \frac{1}{\sqrt{N_{t,x}}}[1,\exp\left(-j2\pi \frac{d_{v}}{\lambda }\sin\theta_{1}^{y}\cos\theta_{1}^{x}\right),\ldots ,\\ \; & \exp(-j2\pi \left(N_{t,x}-1\right)\frac{d_{v}}{\lambda }\sin\theta_{N_{t,x}-1}^{y}\cos\theta_{N_{t,x}-1}^{x})]\in C^{1\times N_{t,x}} \end{array}$$

(13) $$\begin{array}{cc} v^{y}= & \frac{1}{\sqrt{N_{t,y}}}[1,\exp\left(-j2\pi \frac{d_{v}}{\lambda }\cos\theta_{1}^{y}\right),\ldots ,\\ \; & \exp\left(-j2\pi \left(N_{t,y}-1\right)\frac{d_{v}}{\lambda }\cos\theta_{N_{t,y}-1}^{y}\right)]\in C^{1\times N_{t,y}} \end{array}$$

where λ denotes the wavelength, $d_{v}$ represents the element space of the antenna array. *M* represents the number of antenna elements. $N_{t,x}$, $N_{t,y}$ denote the number of antenna elements along *x* axis and *y* axis, respectively.

Doppler frequency shift includes the frequency shift brought by satellite and terrestrial terminals. Assuming that the IoT UT is not moving, the frequency shift $f=f_{sat}$. According to [33], the frequency shift of different paths can be assumed the same values, $\varepsilon_{l,k}^{LOS}=\varepsilon_{l,k,m}^{NLOS}$. Because the distance between the two receiving antennas of the IoT terminal is far less than the height of the satellite, the frequency shift caused by the same satellite to the two antennas of the IoT terminal can be regarded as the same value, $\varepsilon_{l}=\varepsilon_{l}^{LOS}=\varepsilon_{l,k}^{LOS}$. Therefore, Formulas (9) and (10) can be rewritten as

(14) $$\begin{array}{c} h_{l,k}^{LOS}\left(n\right)=\sqrt{\frac{K_{l,k}}{K_{l,k}+1}}\exp\left[j2\pi \left(\varepsilon_{l}\frac{n}{N}-j2\pi f_{c}\tau_{l,k}^{LOS}\right)\right]v_{l,k}^{LOS} \end{array}$$

(15) $$\begin{array}{c} h_{l,k}^{NLOS}\left(n\right)=\sqrt{\frac{1}{K_{l,k}+1}}\sum_{m=1}^{M_{l,k}}g_{l,k,m}\exp\left[j2\pi \left(\varepsilon_{l}\frac{n}{N}-j2\pi f_{c}\tau_{l}\right)\right]v_{l,k}^{LOS} \end{array}$$

## 3. Proposed Scheme

### 3.1. Synchronization Scheme

PSS and SSS, which have good cross-correlation and autocorrelation properties, are adopted for synchronization. The PSS and SSS are located on SS/PBCH Block, which is defined in [31]. PSS is adopted to estimate delay and coarse doppler offset estimation. PSS and SSS are used to fine doppler offset estimation. As the satellite adopts the staring mode and the terminal adopts the reflector antenna, the interference of another satellite can be ignored. The received signal of antenna 1 can be simplified as

(16) $$\begin{array}{c} y_{1}\left(n\right)=G_{1}x_{1}\left(n\right)\exp\left(j2\pi n\frac{\varepsilon_{1}}{N}\right)\exp\left(-j2\pi f_{c}\tau_{1}\right)+z_{1}\left(n\right) \end{array}$$

where $G_{1}$ represents the complex gain. $\tau_{1}$ denotes the delay from satellite 1 to antenna 1. In order to estimate the large frequency shift of satellite, the two-dimensional search method is adopted for time synchronization and coarse frequency shift estimation. Multiply the received signal by the conjugate of the local PSS and perform Fourier transform to obtain

(17) $$\begin{array}{cc} R_{1}\left(n,k\right)= & \sum_{m=0}^{N_{1}-1}y_{1}\left(n+m\right)s_{pss}^{*}\left(m\right)\exp\left(-j2\pi \frac{m}{N_{1}}k\right)\\ = & \sum_{m=0}^{N_{1}-1}\left[G_{1}x_{1}\left(n+m\right)s_{pss}^{*}\left(m\right)\exp\left(j2\pi m\frac{\varepsilon_{1}}{N}\right)\right.\\ \; & \left.\exp\left(-j2\pi f_{c}\tau_{1}\right)\exp\left(-j2\pi \frac{m}{N_{1}}k\right)+\psi_{1}\right]\\ = & \sum_{m=0}^{N_{1}-1}\left[G_{1}x_{1}\left(m\right)s_{pss}^{*}\left(m\right)\exp\left(-j2\pi \frac{m}{N}\left(k\frac{N}{N_{1}}-\varepsilon_{1}\right)\right)\right.\\ \; & \left.\exp\left(-j2\pi f_{c}\tau_{1}\right)+\psi_{1}\left(n\right)\right] \end{array}$$

$R_{1}\left(n,k\right)$ denotes the *k*-th Fourier transform value obtained from FFT and cross-correlation sequences, which are calculated from the *n*-th point of the received sequence, where $k=0,1,\ldots ,N_{1}-1$ represents the number of Fourier transform points. $s_{pss}\left(n\right)$ represents the local PSS. *N* denotes the number of FFT points modulated by OFDM. $N_{1}$ represents the Fourier transform points, $N_{1}>N$. $\psi_{1}\left(n\right)=\sum_{m=0}^{N_{1}-1}z_{1}\left(n+m\right)s_{pss}^{*}\left(m\right)\exp\left(-j2\pi \frac{m}{N_{1}}k\right)$ represents the influence of noise on synchronization algorithm. Then, the estimated delay is given by

(18) $$\begin{array}{c} {\hat{n}}_{1}=\underset{n}{\arg\max}\left\{\underset{k}{\arg\max}R_{1}\left(n,k\right)\right\} \end{array}$$

where ${\hat{n}}_{1}$ denotes the estimated delay. In (18), slide in the frequency domain to search for the peak and then slide in the time domain to search for the peak. It can effectively resist the influence of the large frequency shift. When the estimated delay ${\hat{n}}_{1}$ is accurate, bring ${\hat{n}}_{1}$ into (17), (17) can be rewritten as

(19) $$\begin{array}{cc} R_{1}\left({\hat{n}}_{1},k\right)= & \sum_{m=0}^{N_{1}-1}\left[G_{1}{\left|s_{pss}\left(m\right)\right|}^{2}\exp\left(-j2\pi \frac{m}{N}\left(k\frac{N}{N_{1}}-\varepsilon_{1}\right)\right)+\psi_{1}\left(n\right)\right] \end{array}$$

When the value of $\left(kN/N_{1}-\varepsilon_{1}\right)$ reaches its minimum value, the $R_{1}\left({\hat{n}}_{1},k\right)$ can obtain the maximum value. Thus, the estimated normalized frequency can be expressed as

(20) $$\begin{array}{c} {\hat{k}}_{1}=\underset{n}{\arg\max}R_{1}\left({\hat{n}}_{1},k\right) \end{array}$$

(21) $$\begin{array}{c} \varepsilon_{c}={\hat{k}}_{1}\frac{N}{N_{1}} \end{array}$$

The accuracy of $\varepsilon_{c}$ is affected by the Fourier transform points $N_{1}$ in Equation (17). The estimated frequency offset $\varepsilon_{c}$ is used to compensate for the signal. Then, the residual frequency offset is estimated by the fractional frequency offset estimation. Take out the signal corresponding to the local PSS mapping position in the received signal, which can be expressed as

(22) $$\begin{array}{c} {y^{\prime }}_{1}\left(n\right)=G_{1}s_{pss}\left(n\right)e^{j2\pi n\frac{\varepsilon_{r}}{N}}+z_{1}\left(n\right) \end{array}$$

where $G_{1}$ denotes the complex gain. $s_{pss}\left(n\right)$ represents the local PSS. $\varepsilon_{r}$ denotes the residual frequency shift. *N* represents the number of Fourier transform points in OFDM modulation. $z_{1}\left(n\right)$ denotes the additive Gaussian white noise. The conjugate of the local PSS and ${y^{\prime }}_{1}\left(n\right)$ are multiplied to construct $r_{1}\left(n\right)$. $r_{1}\left(n\right)$ can be expressed as

(23) $$\begin{array}{cc} r_{1}\left(n\right) & =s_{pss}^{*}\left(n\right)\cdot {y^{\prime }}_{1}\left(n\right)=s_{pss}^{*}\left(n\right)\left[G_{1}s_{pss}\left(n\right)e^{j2\pi n\frac{\varepsilon_{r}}{N}}+z_{1}\left(n\right)\right]\\ & =G_{1}{\left|s_{pss}\left(n\right)\right|}^{2}e^{j2\pi n\frac{\varepsilon_{r}}{N}}+{\psi^{\prime }}_{1}\left(n\right) \end{array}$$

where ${\psi^{\prime }}_{1}\left(n\right)=s_{pss}^{*}\left(n\right)z_{1}\left(n\right)$. Divide $r_{1}\left(n\right)$ into two part sequences with the same length. Ignore the influence of the noise, the correlation between the two sequences can be expressed as

(24) $$\begin{array}{cc} R_{2} & =\sum_{n=0}^{N_{2}/2-1}{r_{1}}^{*}\left(n\right)r_{1}\left(n+N_{2}/2\right)\\ \; & =\sum_{n=0}^{N_{2}/2-1}{G_{1}}^{*}{\left|s_{pss}\left(n\right)\right|}^{2}e^{-j2\pi n\frac{\varepsilon_{r}}{N}}G_{1}{\left|s_{pss}\left(n+N_{2}/2\right)\right|}^{2}e^{j2\pi \left(n+N_{2}/2\right)\frac{\varepsilon_{r}}{N}}\\ \; & =e^{j\pi N_{2}\frac{\varepsilon_{r}}{N}}\sum_{n=0}^{N_{2}/2-1}{\left|G_{1}\right|}^{2}{\left|s_{pss}\left(n\right)\right|}^{2}{\left|s_{pss}\left(n+N_{2}/2\right)\right|}^{2} \end{array}$$

where $N_{2}$ is the length of $r_{1}\left(n\right)$. The estimated frequency offset is given by

(25) $$\begin{array}{c} {\hat{\varepsilon }}_{d}=\frac{angle(R_{2})}{\pi }\frac{N}{N_{2}} \end{array}$$

where ${\hat{\varepsilon }}_{d}\in \left(-N/N_{2},N/N_{2}\right)$. As the $N_{1}>N_{2}$, the residual frequency offset is $\varepsilon_{r}<N/N2$. Therefore, this method can estimate residual frequency offset.

To improve the accuracy of the frequency offset estimation, PSS and SSS are used to estimate the frequency offset further. Construct $r_{pss}$ and $r_{sss}$ as

(26) $$\begin{array}{c} r_{pss}\left(n\right)=s_{pss}^{*}\left(n\right)\cdot {y^{\prime }}_{1}\left(n\right)\\ r_{sss}\left(n\right)=s_{sss}^{*}\left(n\right)\cdot {y^{\prime }}_{1}\left(n+L\right) \end{array}$$

where $\cdot {y^{\prime }}_{1}\left(n\right))$ and $\cdot {y^{\prime }}_{1}\left(n+L\right)$ represent the extracted PSS and SSS corresponding to the received signal. *L* represents the distance between the mapping positions of PSS and SSS. $s_{pss}$ and $s_{sss}$ denote the local PSS and SSS, respectively. Correlate $r_{pss}$ and $r_{sss}$ to obtain

(27) $$\begin{array}{cc} R_{3} & =\sum_{n=0}^{N_{2}-1}r_{pss}^{*}\left(n\right)r_{sss}\left(n\right)\\ \; & =\sum_{n=0}^{N_{2}-1}G_{1}^{*}{\left|s_{pss}\left(n\right)\right|}^{2}e^{-j2\pi n\frac{\varepsilon_{r}}{N}}G_{1}^{}{\left|s_{sss}\left(n\right)\right|}^{2}e^{j2\pi \left(n+L\right)\frac{\varepsilon_{r}}{N}}\\ \; & =e^{j2\pi L\frac{\varepsilon_{r}}{N}}\sum_{n=0}^{N_{2}-1}{\left|G_{1}\right|}^{2}{\left|s_{pss}\left(n\right)\right|}^{2}{\left|s_{sss}\left(n\right)\right|}^{2} \end{array}$$

where $N_{2}$ denotes the length of $r_{pss}$, which is also the length of $r_{sss}$. The estimated frequency offset is given by

(28) $$\begin{array}{c} {\hat{\varepsilon }}_{d}=\frac{angle(R_{3})}{\pi }\frac{N}{2L} \end{array}$$

where the estimated frequency offset range is ${\hat{\varepsilon }}_{d}\in \left(-N/2L,N/2L\right)$. Therefore, the $N_{1}$ in (17) could not be less than N/2L.

With the two signals streams received from two antennas of the IoT UT, the frequency offset is estimated according to (17)–(28) and compensated, respectively.

### 3.2. Power Control Scheme

**Figure 4:**
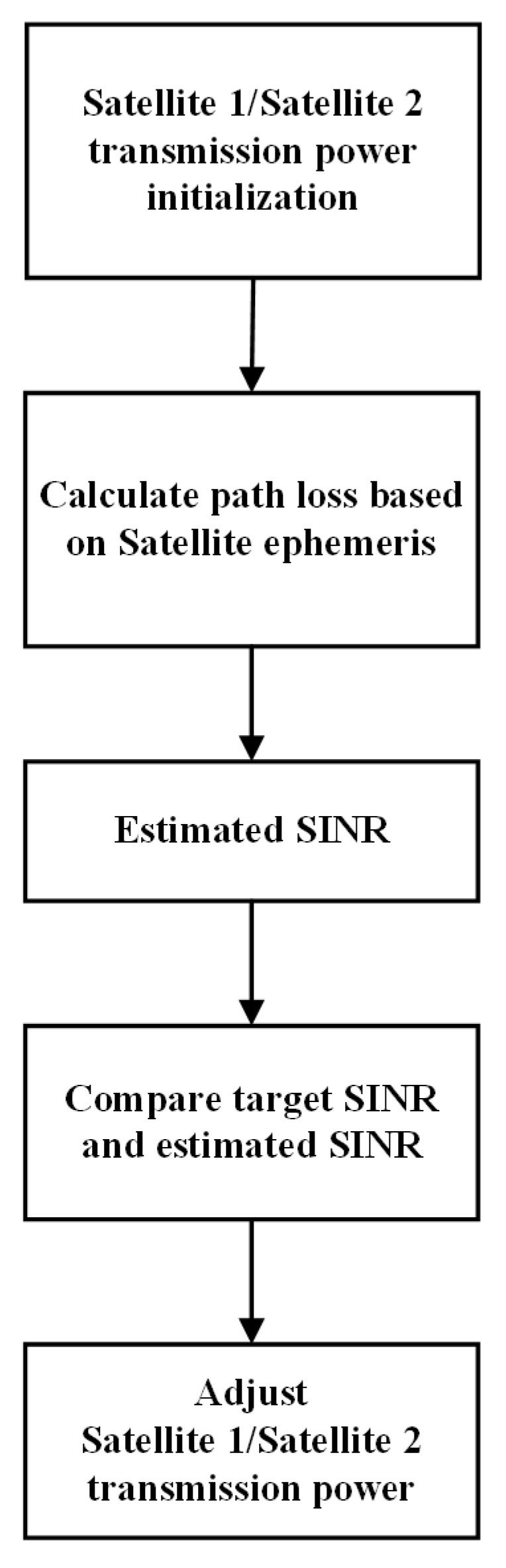
Diagram of open-loop power control.

**Figure 5:**
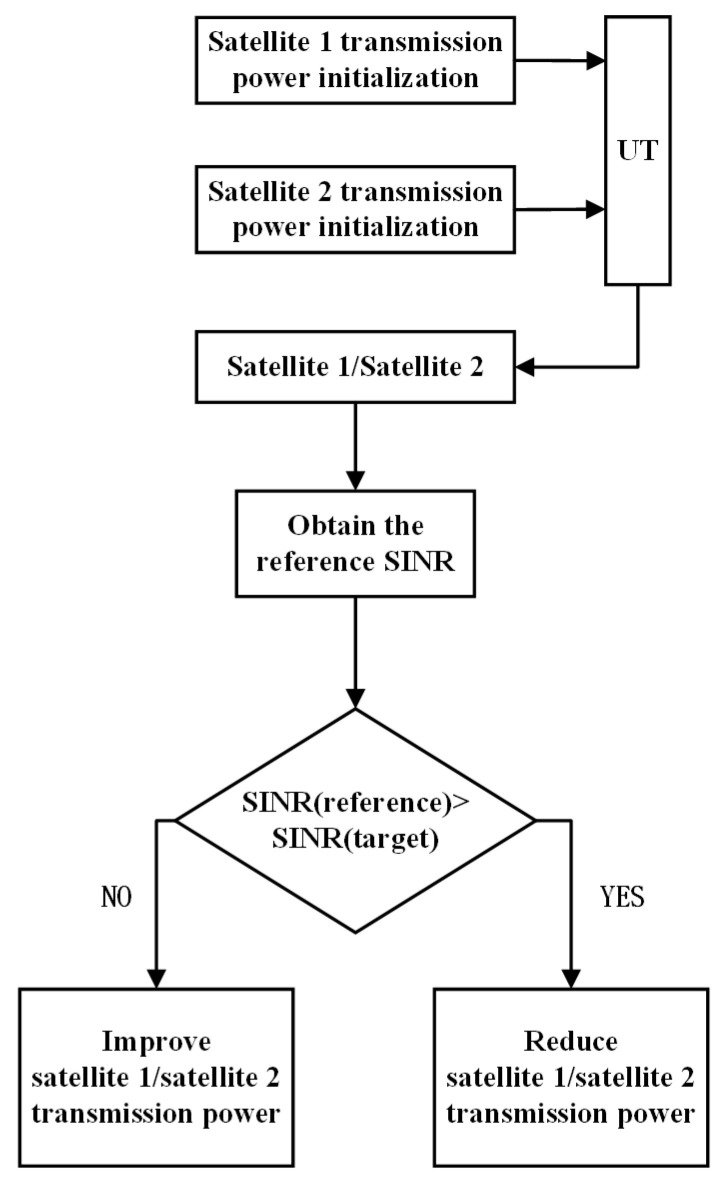
Diagram of closed-loop power control.

Power control methods include open-loop power control and closed-loop power control. Open-loop power control can provide a coarse estimate of the transmission power and does not require the receiver to provide feedback, which is simple to realize and convenient for engineering applications. Closed-loop power control needs feedback information from the receiver with higher complexity and better performance compared with open-loop power control. The process of these power control methods is shown in Figure 4 and Figure 5.

#### 3.2.1. Closed-Loop Power Control

As shown in Figure 5, the transmission power of satellite 1 and satellite 2 should be initialized at first. The initialization transmission power of satellite 1 and satellite 2 can be expressed as

(29) $$\begin{array}{c} P_{sat1}^{1}=\min\left(P_{sat1}^{\max},P_{sat1}^{0}+\alpha \cdot PL_{sat1}+10\log_{10}M\right)\\ P_{sat2}^{1}=\min\left(P_{sat2}^{\max},P_{sat2}^{0}+\alpha \cdot PL_{sat2}+10\log_{10}M\right) \end{array}$$

where $P_{sat1}^{\max},P_{sat2}^{\max}$ denote the maximum power that satellite 1 and satellite 2 can transmit. $P_{sat1}^{0},P_{sat2}^{0}$ represent the path loss (PL) of satellite 1 and satellite 2. α denotes the path loss compensation factor. *M* is the number of resource blocks (RB) allocated. $P^{0}$ can be expressed as

(30) $$\begin{array}{c} P_{sat1}^{0}=\alpha \cdot \left(SINR_{sat1}^{0}+P_{n}\right)+\left(1-\alpha \right)\cdot \left(P_{sat1}^{\max}-10log_{10}M_{0}\right)\\ P_{sat2}^{0}=\alpha \cdot \left(SINR_{sat2}^{0}+P_{n}\right)+\left(1-\alpha \right)\cdot \left(P_{sat2}^{\max}-10log_{10}M_{0}\right) \end{array}$$

where $SINR_{sat1}^{0},SINR_{sat2}^{0}$ represent the target signal-to-interference-noise ratio (SINR). $P_{n}$ is the noise power of each RB. When α=1, (30) can be rewritten as

(31) $$\begin{array}{c} P_{sat1}^{0}=\left(SINR_{sat1}^{0}+P_{n}\right)\\ P_{sat2}^{0}=\left(SINR_{sat2}^{0}+P_{n}\right) \end{array}$$

Assuming the initial time is $t_{0}$, and the power control adjustment cycle is *T*. At time $t_{0}+T$, the transmission power of satellite 1 and satellite 2 $P_{sat1}^{2},P_{sat2}^{2}$ can be represented as

(32) $$\begin{array}{c} P_{sat1}^{2}=P_{sat1}^{1}-d_{sat1}^{1}\\ P_{sat2}^{2}=P_{sat2}^{1}-d_{sat2}^{1} \end{array}$$

where $d_{sat1}^{1},d_{sat2}^{1}$ denote the power variation value of satellite 1 and satellite 2, which is given by

(33) $$\begin{array}{c} d_{sat1}^{1}=SINR_{sat1}^{ref1}-SINR_{sat1}^{0}\\ d_{sat2}^{1}=SINR_{sat2}^{ref1}-SINR_{sat2}^{0} \end{array}$$

$SINR_{sat1}^{ref1},SINR_{sat2}^{ref1}$ denote the reference SINR of satellite 1 and satellite 2 through the feedback information of UT at time $t_{0}+T$. $SINR_{sat1}^{0},SINR_{sat2}^{0}$ represent the target SINR of satellite1 and satellite 2, respectively.

The closed-loop power control process is as follows:

Step1: Initialize the transmission power of satellite 1 and satellite 2.

Step2: Obtain reference SINR of satellite 1 and satellite 2 through feedback information of UT at time $t_{0}+T$. Calculate the power variation value of satellite 1 and satellite 2 according to (33).

Step3: Calculate transmission power of satellite 1 and satellite 2 at time $t_{0}+T$ according to (32).

Step4: For each subsequent adjustment cycle *T*, according to step 2 and step 3, update the power change value and transmission power.

#### 3.2.2. Open-Loop Power Control

In open-loop power control, the power is adjusted by calculating the PL. In order to obtain the PL, we need to calculate the position of the satellite in the orbit according to the six elements of the orbit so as to estimate the path loss of the satellite to the ground station. Select the Earth inertial coordinate system (ECI) as the reference system to analyze the various characteristics of each parameter. Based on the Walker constellation [32], the distribution of satellites in the constellation can be expressed as N/P/F, where *P* denotes the total number of orbits contained in the constellation, *F* represents the phase factor of the constellation and *S* denotes the number of satellites in each orbit. N=S×P is the total number of satellites in the constellation. The phase offset between the nearest two satellites between adjacent orbits is determined by 2πF/N, while the value range of *F* is [0,P−1]. According to the spatial–geometric relationship, the position of the *k*-th satellite in the *i*-th orbit of the constellation can be represented by

(34) $$\begin{array}{c} \begin{array}{c} x_{ik}\left(t\right)=-R\cos\theta \sin\left(\frac{2\pi i}{P}\right)\sin\left[\omega t+2\pi \left(\frac{k}{S}+\frac{iF}{PS}\right)\right]+\\ R\cos\left(\frac{2\pi i}{P}\right)\cos\left[\omega t+2\pi \left(\frac{k}{S}+\frac{iF}{PS}\right)\right]\\ y_{ik}\left(t\right)=R\cos\theta \cos\left(\frac{2\pi i}{P}\right)\sin\left[\omega t+2\pi \left(\frac{k}{S}+\frac{iF}{PS}\right)\right]+\\ R\sin\left(\frac{2\pi i}{P}\right)\cos\left[\omega t+2\pi \left(\frac{k}{S}+\frac{iF}{PS}\right)\right]\\ z_{ik}\left(t\right)=R\sin\theta \sin\left[\omega t+2\pi \left(\frac{k}{S}+\frac{iF}{PS}\right)\right] \end{array} \end{array}$$

where 0≤i≤P−1,0≤k≤S−1. *R* denotes the distance between the satellites and the earth’s center, ω is the orbital angular velocity of the satellites and θ represents the orbital inclination of the satellites. The six orbital elements include semi major axis, eccentricity, inclination, the longitude of ascending node, the argument of periapsis and true anomaly. When the orbit is a circular orbit, the argument of periapsis and true perigee angle can be merged into the argument of latitude, and the eccentricity is zero. (34) can be expressed by the satellite orbital elements as

(35) $$\begin{array}{c} \begin{array}{c} x_{ik}\left(t\right)=-R\cos\theta \sin\Omega \sin\left(\alpha +\varphi \right)+R\cos\Omega \cos\left(\alpha +\varphi \right)\\ y_{ik}\left(t\right)=-R\cos\theta \cos\Omega \sin\left(\alpha +\varphi \right)+R\sin\Omega \cos\left(\alpha +\varphi \right)\\ z_{ik}\left(t\right)=R\sin\theta \sin\left(\alpha +\varphi \right) \end{array} \end{array}$$

where *R* represents semi major axis, θ denotes the inclination. Ω is the longitude of ascending node. α is the argument of periapsis, and ϕ denotes the true anomaly. Set the coordinates of ground terminal $\left[x_{0}\left(t\right),y_{0}\left(t\right),z_{0}\left(t\right)\right]$, the distance between satellites and ground terminal can be expressed

(36) $$\begin{array}{c} d_{k}=\sqrt{{\left(x_{k}\left(t\right)-x_{0}\left(t\right)\right)}^{2}+{\left(y_{k}\left(t\right)-y_{0}\left(t\right)\right)}^{2}+{\left(z_{k}\left(t\right)-z_{0}\left(t\right)\right)}^{2}} \end{array}$$

According to [33], PL can be modeled by

(37) $$\begin{array}{c} PL_{b}=FSPL\left(d,f_{c}\right)+SF+CL\left(\alpha ,f_{c}\right) \end{array}$$

where $FSPL(d,f_{c})$ denotes the free-space path loss (FSPL) in dB, which is given by

(38) $$\begin{array}{c} FSPL\left(d,f_{c}\right)=32.45+20\log_{10}\left(f_{c}\right)+20\log_{10}\left(d\right) \end{array}$$

*d* is the distance between the satellite and ground terminal, $f_{c}$ is the carrier frequency. SF denotes the shadow fading, $CL(\alpha ,f_{c})$ represents the cluster loss.

The open-loop power control process is described as follows:

Step 1: Determine the target SINR of satellite 1 and satellite 2, calculate the transmission power $P_{sat1}^{1},P_{sat2}^{1}$ at the initial time according to the ephemeris information and Formulas (29)–(38). $P_{sat1}^{1},P_{sat2}^{1}$ represent the transmission power of satellite 1 and satellite 2 at the initial time.

Step 2: Obtain ephemeris information at time $t_{0}+T$. Calculate the two satellites’ transmission power at time $t_{0}+T$ according to Formulas (29)–(38), where *T* is the power adjustment cycle.

Step 3: Set the transmission power $P_{sat1}^{2},P_{sat2}^{2}$ from $t_{0}+T$ to $t_{0}+2T$. Calculate and update the transmit power according to Step 2 at the time $t_{0}+2T$.

Step 4: Set the transmission power $P_{sat1}^{N-1},P_{sat2}^{N-1}$ from $t_{0}+\left(N-1\right)T$ to $t_{0}+NT$, calculate and update the transmission power of satellite 1 and satellite 2 $P_{sat1}^{N},P_{sat2}^{N}$ at the time $t_{0}+NT$ according to Steps 2 and 3.

## 4. Results

In this section, we will evaluate the proposed scheme via a simulation. The satellite orbit height of the OneWeb system is 1200 km, each orbit plane contains 40 satellites, the included angle between each satellite is 9° and the orbit inclination of the OneWeb, SpaceX and Telesat is between 37.4° and 87.9° [34]. When the orbit height becomes higher, the frequency offset brought by the satellite will be reduced, which is conducive to reducing the adverse impact of the frequency offset on the system. Therefore, we consider the height of the LEO satellites as 1200 km. The satellite orbit inclination is 86.4°. The two satellites are located on the same orbital plane with an included angle of 9°. The time period for each satellite that can connect with the IoT UT is 16 min. The terminal antenna adopts the reflector antenna defined in [35]. The normalized antenna radiation pattern can be expressed as

(39) $$G\left(\theta \right)=\left\{\begin{array}{c} 1,\theta =0^{\circ }\\ 4{\left|\frac{J_{1}\left(ka\sin\theta \right)}{ka\sin\theta }\right|}^{2}{,0}^{\circ }<\left|\theta \right|\leq 90^{\circ } \end{array}\right.$$

where $J_{1}\left(x\right)$ denotes the first kind of first-order Bessel function, *a* is the radius of the circular hole of the antenna, k=2πf/c represents the wave number, *f* is the central frequency of the system and *c* denotes the speed of light in vacuum. θ represents the angle measured from the bore sight of the antenna’s main beam. Table 1 shows the link parameters of the system. A Rician channel is adopted, and the Rician factor is 13.3 dB.

**Figure 6:**
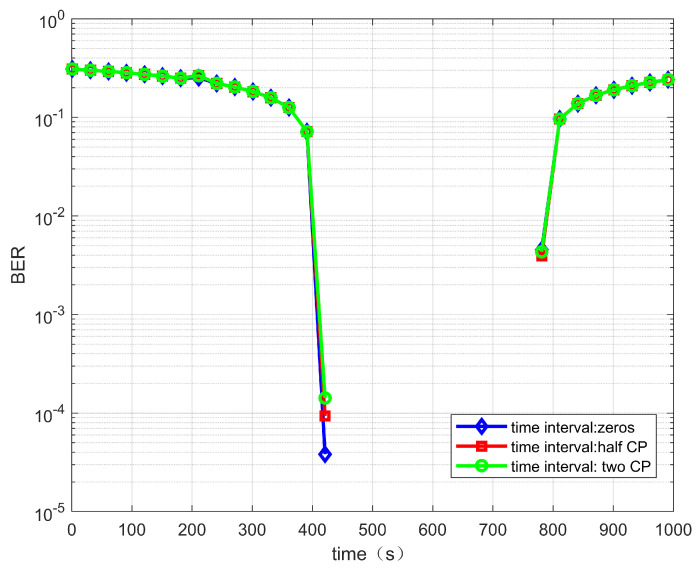
BER performance versus time for the link between satellite 1 and IoT terminal.

**Figure 7:**
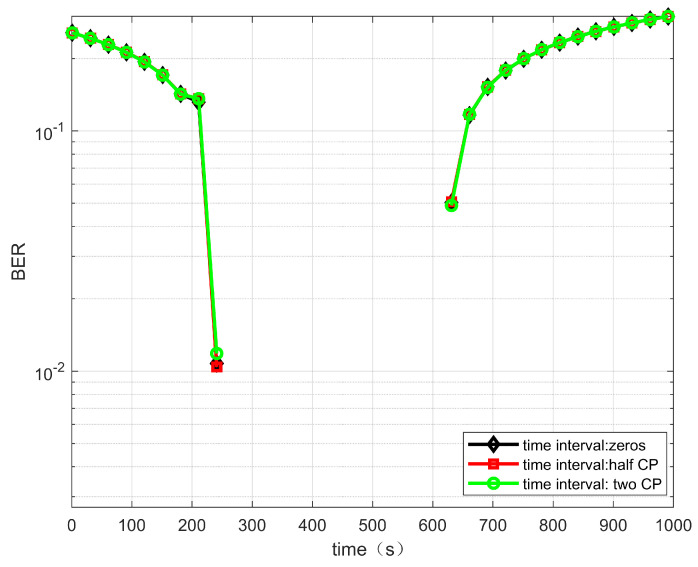
BER performance versus time for the link between satellite 2 and IoT terminal.

When the transmitted signals from two satellites do not arrive at the terminal at the same time, the transmitted signals from different satellites will cause interference and affect the demodulation performance. In the case of an ideal frequency offset compensation, the BER performance of the dual-stream signals arriving at the terminal with different time intervals is analyzed. Figure 6 and Figure 7 show the case of no time interval, a half CP time interval and two CP time intervals. When the reflector antenna is used, the mutual interference between the two satellites of the signals is small. Thus, different time intervals have little effect on the demodulation performance of the system.

**Figure 8:**
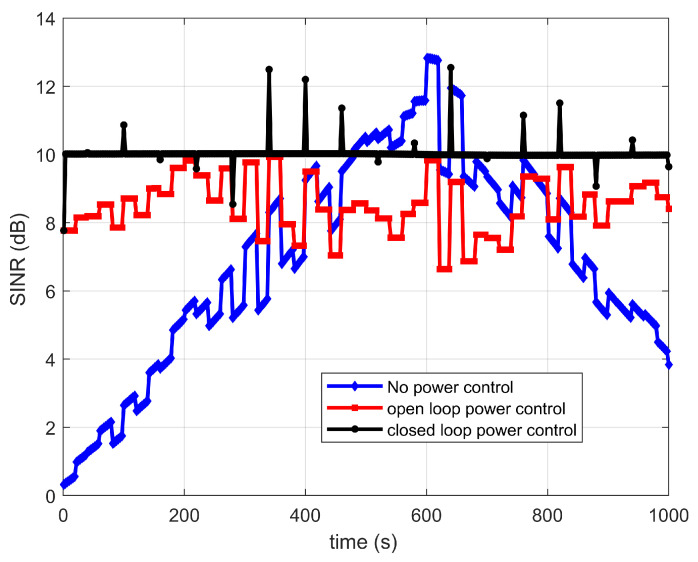
SINR for link between satellite 1 and IoT terminal versus time.

**Figure 9:**
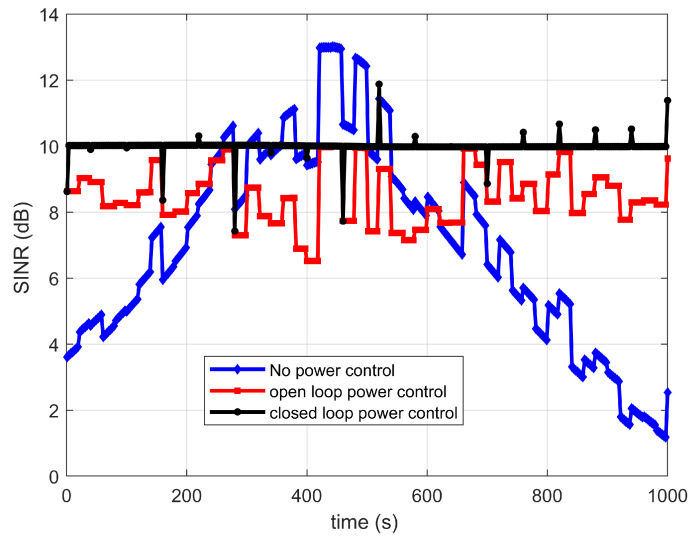
SINR for link between satellite 2 and IoT terminal versus time.

In order to verify the power control performance of different power control schemes, the dual transmission with no power control, open-loop power control and closed-loop power control are investigated. The shadow fading is considered for suburban and rural scenarios, and the variance value is given by [33]. The target SINR is set to 7 dB. The power adjustment cycle is set to 1 s. Perfect synchronization is assumed. Figure 8 and Figure 9 show the SINR versus time curve under different power control schemes. Compared with the no power control scheme, the SINR with power control schemes are more stable. In addition, the closed-loop power control scheme is more stable than the open-loop power control. However, the closed-loop power control scheme needs the feedback information from the terminal, and it will increase the system complexity. Thus, in the following analysis, the open-loop power control scheme is assumed to be adopted.

**Figure 10:**
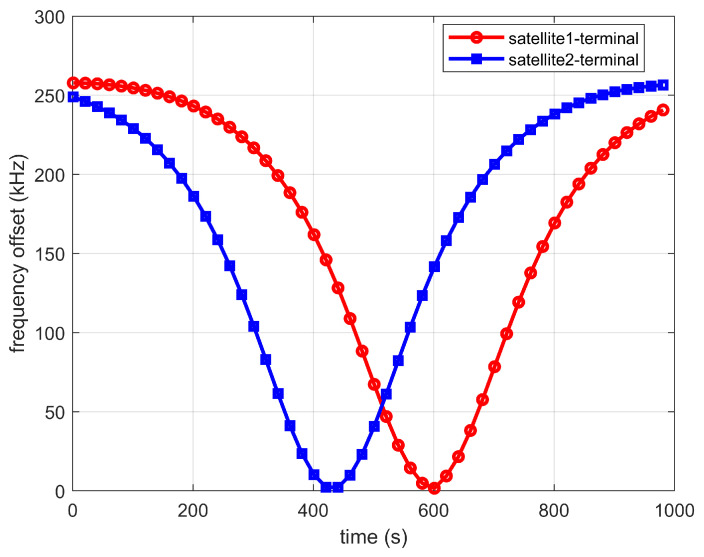
Dynamic characteristics of frequency offset along with time.

**Figure 11:**
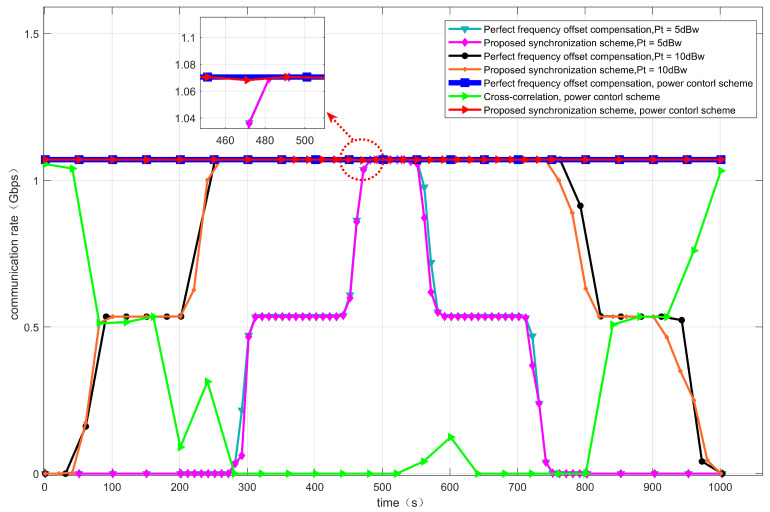
Terminal communication rate with fixed transmit power and power control scheme.

Figure 10 shows the dynamic characteristic of the frequency offset experienced by the two satellites. It can be seen that the frequency offset caused by the satellites is different. At the receiver of the IoT terminal, the frequency offset caused by different satellites should be compensated individually. Figure 11 shows the comparison of the terminal communication rate under a perfect frequency offset compensation and the proposed synchronization schemes. Moreover, the effects of the power control scheme are also investigated with the proposed synchronization scheme. When the transmission power is 5 dbw, the communication rate can hardly be maintained above 1 Gbps. When the transmission power increases to 10 dbw, the communication rate can be maintained above 1 Gbps for about ten minutes. When adopting the power control scheme, the transmit power of two satellites will change according to the adopted power control scheme, and the SNR can maintain a relatively stable range. Thus, the communication rate can be maintained above 1 Gbps all the time. We compare the cross-correlation algorithm in [36] with the method in this paper, as shown in Figure 11.The simulation results show that under the SINR, modulation and coding rate, the cross-correlation algorithm has poor performance in the frequency offset estimation and compensation, so the communication rate is low. Compared with the perfect frequency offset compensation, the simulation results show that under the proposed synchronization scheme, the communication rate will fluctuate slightly, as shown in the enlarged part of the red circle in Figure 11, which is caused by the frequency offset estimation error. The overall communication rate can still be maintained above 1 Gbps.

## 5. Conclusions

In this paper, the dual-stream transmission and power control for multiple satellites-assisted IoT networks are investigated to improve the data rate of IoT terminals. In order to resist the influence of high-moving LEO satellites, a multi-satellites synchronization scheme is proposed to compensate for the frequency offset caused by different satellites. Moreover, the downlink power control scheme is investigated to keep the data rate stable. In addition, in the hardware implementation, because the proposed synchronization scheme adopts FFT, it is necessary to select the appropriate number of FFT points to reduce the complexity of the algorithm and facilitate the hardware implementation. The simulation results show that the proposed scheme can effectively resist the frequency offset of LEO satellites, achieve good performance and the communication rate can reach more than 1 Gbps for a single IoT terminal.

## Figures and Tables

**Table 1:** System Parameters.

Parameter	Value
Carrier frequency	18 GHz
Bandwidth	400 MHz
Satellites antenna gain (dB)	23
User’s antenna gain (dB)	39.7
Code	turbo
Code rate	600/1024
Modulation	8APSK

## Data Availability

Not applicable.
